# Draft Genome Sequence of a Highly Heterozygous Yeast Strain from the Metschnikowia pulcherrima Subclade, UCD127

**DOI:** 10.1128/genomeA.00550-18

**Published:** 2018-06-21

**Authors:** Anjan Venkatesh, Anthony L. Murray, Adrian B. Boyle, Lisa Quinn Farrington, Timothy J. Maher, Peadar Ó’Gaora, Kenneth H. Wolfe, Caoimhe E. O’Brien, Geraldine Butler

**Affiliations:** a School of Biomedical and Biomolecular Sciences, Conway Institute, University College Dublin, Dublin, Ireland; b School of Medicine, Conway Institute, University College Dublin, Dublin, Ireland

## Abstract

Metschnikowia strain UCD127 was isolated from soil in Ireland and sequenced. It is a highly heterozygous diploid strain with 385,000 single nucleotide polymorphisms (SNPs). Its ribosomal DNA has the highest similarity to that of M. chrysoperlae, but its *ACT1* and *TEF1* loci and mitochondrial genome show affinity to those of M. fructicola, whose genome is significantly larger.

## GENOME ANNOUNCEMENT

Metschnikowia spp. are yeasts that make characteristic needle-shaped spores. One of its subclades contains nine very closely related species, of which the best known is M. pulcherrima (1). The subclade also includes M. fructicola, M. chrysoperlae, and M. zizyphicola, among others (2–4). Species in this subclade are used commercially as biocontrol agents to prevent fruit spoilage, because they can kill molds (5, 6). However, because they are autogamous, defining species in this subclade has been based solely on molecular data and not reproductive isolation (7). A genome sequence is available for M. fructicola (5) but not for any other species in the subclade.

We isolated strain UCD127 from soil in a forest in Ireland (global positioning system [GPS] coordinates, N53.405750, W7.041967). It was cultured on yeast-peptone-dextrose (YPD) nutrient agar plates containing chloramphenicol (3% [wt/vol]) and carbenicillin (10% [wt/vol]) at 30°C. Sequencing the internal transcribed spacer (ITS) region suggested affinity to the M. pulcherrima subclade. Total genomic DNA was extracted with phenol-chloroform, and purified using a DNA Clean & Concentrator-25 kit (Zymo Research). Genomic DNA was sequenced by BGI Tech Solutions (Illumina HiSeq 4000) from libraries containing fragments of 170 to 800 bp. A total of 6.7 million paired-end reads (2 × 150 bp) were generated. Low-quality reads were trimmed using Skewer v0.2.1 (8).

The genome was assembled separately using SPAdes (9) and dipSPAdes v3.11.1 (10). QUAST v4.6 was used to assess assembly quality (11). Since the dipSPAdes assembly had substantially fewer contigs (33 contigs versus 7,594 contigs > 1 kb) and a higher *N*_50_ value (151 kb versus 2.5 kb), the genome was hypothesized to be highly heterozygous. The total assembly sizes were 16.1 Mb from dipSPAdes and 17.1 Mb from SPAdes, ignoring contigs of <1 kb. Analysis of variants was carried out using BWA (12) and SAMtools v1.4 (13) to map reads to the dipSPAdes assembly. A total of 385,486 SNPs and 45,673 indels were found. Histogram analysis of biallelic SNP frequencies confirmed that the genome is diploid.

Annotation of the dipSPAdes assembly using AUGUSTUS (14) predicted 5,807 protein-coding genes, which is in line with those of other ascomycete yeasts but much fewer than the 9,674 predicted in the 26-Mb M. fructicola genome (5). tRNAscan-SE identified 173 tRNA genes, including two genes for tRNA^Ser^(CAG) with characteristic G_33_ and G_73_ positions, indicating that UCD127 translates CUG codons as serine, as expected for this genus (15).

UCD127 is in the M. pulcherrima subclade, but its exact species designation is uncertain. Phylogenetic analysis of the D1/D2 rDNA region clustered it with M. chrysoperlae (1 difference in 516 bp) and the unnamed strain NRRL Y-6148 (2 differences) rather than M. pulcherrima (5 differences) or M. fructicola (7 differences). However, the *ACT1* and *TEF1* sequences of UCD127 show a closer relationship to M. fructicola and M. pulcherrima than to M. chrysoperlae, and the mitochondrial genome has 99% sequence identity with M. fructicola (mitochondrial DNAs [mtDNAs] of the other species have not sequenced).

### Accession number(s).

This whole-genome shotgun project has been deposited in DDBJ/ENA/GenBank under accession no. QBLL00000000. The version described in this paper is the first version, QBLL01000000.
